# Treatise on Dislocations of the Shoulder

**Published:** 1849

**Authors:** 


### ARTICLE II.

*Treatise on dislocations of the shoulder.* By George O. Jarvis, M. D., author of Lectures on Fractures and Dislocations &c. &c. Together with important cases illustrating the benefits of the adjuster. Compiled by George Kellogg A. M. Derby, New Haven County Conn. Birmingham. 1848.

We find upon our table a small pamphlet of 32 pages, with the above title, indicating as one would suppose, that the reader might expect to find in the body of the work something truly valuable in a "treatise" devoted exclusivly to the consideration of luxations of the "shoulder".

George Kellogg, A. M., the compiler, says.

At the solicitation of many members of the profession, whose opinion I highly esteem, I have been induced, with the consent of the author, to offer to the surgeons of the United States a reprint of this learned and conclusive article, and to add to it a few of the remarkable cases which strikingly attest and exemplify the advantages—the indispensible necessity of the Adjuster. Being the sole manufactuer of that Instrument in America, the value and importance of which are so clearly proved in the following pages, I cannot but hope that while I am, in this way, doing what I can, to promote the usefulness and unfailing success of the whole body of practicing surgeons; they, in their turn, will not be regardless of the claims of humanity upon them, nor of their own professional honor, in these days of quackery, but will encourage, by their influence and example, the universal introduction among them of an apparatus so valuable.

Or in other words, George O. Jarvis M. D. has invented some kind of an instrument, which he calls by the imposing name of the Adjuster, and, as we have reason to believe, judging from the tenor of the pamphlet before us, has chosen to employ his inventive genius and standing as an M. D. for the pecuniary benefit of himself and George Kellogg A. M. "the sole manufacturer of the instrument in America".

Now since it is true that nothing of importance is said in the pamphlet in referance to the symptoms or treatment of dislocations of the shoulder, excepting so far as to condemn the practice of all those who are so unmindful of its merits as not to purchase and use the aforesaid instrument, we are at loss to know why it was published "at the solicitation of many members of the profession." unless, as is possible, the physicians of Connecticut consider themselves justified in employing to the best advantage the peculiar talents for which the people of that state are so justly celebrated, whether it be in the invention and "sole manufacture" of wooden nut megs or Jarvis's adjusters. H

